# Distress and well-being in dentists: performance of a screening tool for assessment

**DOI:** 10.1038/s41405-024-00185-9

**Published:** 2024-01-16

**Authors:** Subha Giri, Colin P. West, Tait Shanafelt, Daniel Satele, Liselotte N. Dyrbye

**Affiliations:** 1 Department of Dental Specialties, Mayo Clinic, Rochester, MN USA; 2 Department of Medicine and Department of Quantitative Health Sciences, Clinic, Rochester, MN USA; 3 Stanford Medicine, Stanford University, Stanford, CA USA; 4 Department of Quantitative Health Sciences, Mayo Clinic, Rochester, MN USA; 5 Department of Medicine, University of Colorado School of Medicine, Aurora, CO USA; 6 Present Address: Department of Quantitative Health Sciences, Mayo Clinic, Rochester, MN USA

**Keywords:** Occupational health, Dental epidemiology, Dental psychology

## Abstract

**Objectives:**

Dentists’ well-being is being challenged today by many factors. However, effective screening tools to assess their distress and well-being are yet to be validated. The present study aims to evaluate the ability of the Well-Being Index (WBI) to identify distress and stratify dentists’ well-being and their likelihood for adverse professional consequences.

**Method and materials:**

A convenience sample of dentists completed a web-based 9-item WBI survey along with other instruments that measured quality of life (QOL), fatigue, burnout, and questions about suicidal ideation, recent dental error, and intent to leave their current job.

**Results:**

A total of 597 dentists completed the survey. The overall mean WBI score was 2.3. The mean WBI score was significantly greater in dentists with low QOL than among dentists without low QOL (4.1 vs 1.6, *p* < 0.001). Dentists with extreme fatigue, burnout, and suicidal ideation had significantly higher mean WBI score than those without distress (all *p* < 0.001). WBI score stratified the dentists’ likelihood of reporting a recent dental error and intent to leave their current job.

**Conclusion:**

The WBI may be a useful screening tool to assess well-being among dentists and identify those in distress and at risk for adverse professional consequences.

## Introduction

Chronic, unmitigated work stress can result in burnout, a syndrome characterized by high emotional exhaustion, high depersonalization (cynicism), and a low sense of personal accomplishment from work [1]. Up to 50% of US physicians experience burnout at a given point in time [2–6]. Physicians who experience burnout are more likely to make medical errors and deliver suboptimal patient care, be involved in malpractice litigation, and reduce their working hours and leave their job than physicians without burnout [7–13]. Relationships have also been found between physician burnout and suicidal ideation and substance use disorder [14–17]. Factors contributing to burnout among physicians include high work hours, work inefficiencies, lack of autonomy, reduced meaning in work, and conflict between work and personal responsibilities [2, 18–20].

While extensive research has been conducted in physicians [21], limited studies have focused on dentists. Available research estimates the global prevalence of burnout among dentists ranges from 7.4–84% [22–24]. This wide range of estimated prevalence reflects variability in study designs and rigor, as well as geographic variability in practice stressors and expectations. The consequences of burnout among dentists have yet to be rigorously delineated but likely include consequences for patients (e.g., dental errors and dental malpractice), employers (e.g., turnover), and dentists themselves. Dentists certainly face a myriad of work stressors, some of which are similar to physicians (e.g., high work hours, work inefficiencies, lack of autonomy, work-home conflict, health care disparities, costs of care, the opioid epidemic, infection control, etc.) with others unique to the dental profession (for example, rapidly developing advances in digital technology, the changing landscape of oral health practice settings) [25]. In a 2021 national study dentists reported at least a moderate stress level at work [26], placing them at risk for burnout and other forms of distress. Preliminary research in dentists suggests that the risk for burnout varies by age, gender, personality, and work hours [27, 28], mirroring studies in physicians [21].

Assessing the prevalence of burnout and other forms of distress among dentists at an individual level as well as at an organizational level may be critical to advancing interventions aimed at reducing work stress and improving the work environment. Studies suggest accurate self-assessment is challenging for healthcare professionals [29]. In a study of 1150 US surgeons, 89% thought their level of well-being was at or above average, including 70% of those with WBI scores in the bottom third. Overall, 49% of the surgeons found seeing their WBI score helpful and 47% intended to make a behavior change directly as a result of the feedback (i.e., transitioning from the precontemplation phase of behavioral change to the contemplation phase) [29]. At the level of an organization, aggregate WBI scores may be useful to organizations wishing to measure dentist well-being and identify subgroups of dentists with worse well-being who may warrant additional resources or intervention. Additionally, longitudinal tracking of WBI scores at an organization level can provide insight into how newly implemented organizational strategies are impacting dentist well-being. Weill Cornell Medicine in New York City is an example of how the WBI can be used to track and respond to healthcare worker well-being using system-level interventions [30].

Most current instruments to measure burnout and other dimensions of well-being (e.g., stress, quality of life, fatigue) and professional life (e.g., meaning in work, satisfaction with work-life integration) only measure a single dimension of distress or well-being. Combining such instruments to holistically assess well-being creates high responder burden and can be cumbersome for organizations to analyze due to separate scoring of each individual instrument. For example, the Maslach Burnout Inventory-Human Services Survey, widely considered the gold standard for measuring burnout [1], contains 22 items. When combined with instruments to assess stress, depression, mental and physical quality of life, and fatigue (each of which is often 4–10 questions in its own right), responder burden increases, potentially threatening response rate.

The Well-Being Index (WBI) is a composite instrument that stratifies multiple dimensions of distress and well-being (stress, burnout, fatigue, depression, mental and physical quality of life, work-life integration, and meaning in work) using 9 items and can be completed in approximately 5 minutes [31]. The validity of the WBI to assess well-being in healthcare professionals (including physicians, nurses, physician assistants, pharmacists, and medical students) has been extensively evaluated. The WBI is simple to score and can stratify risk for distress and the likelihood of positive well-being [32–38]. Notably, WBI scores correlate with the likelihood of adverse professional consequences [32–38]. For example, WBI scores have been shown to effectively stratify the risk of negative professional consequences, such as intent to leave current job and self-reported recent major medical error among healthcare professionals [33–37]. The WBI has also been shown to have strong discriminatory ability for low QOL, burnout, and suicidal ideation among physicians, physician assistants, nurse practitioners, and pharmacists [32–40]. However, the WBI’s ability to identify distress and stratify well-being has not been evaluated among dentists. Therefore, the present study was conducted to evaluate the ability of the WBI to stratify positive well-being (high quality of life [QOL]) and distress (low QOL, extreme fatigue, and burnout) in dentists. It also aims to evaluate the relationship between WBI score and likelihood of dentists reporting a recent major dental error and intent to leave their current job.

## Method and materials

### Participants

Heartland Dental, a nation-wide dental service organization that provides non-clinical support to dentists, sought to validate the WBI in dentistry. With digital newsletters and three e-mail communications, they promoted the use of the web-based version of the WBI among approximately 1200 Heartland Dental supported dentists, asking them to complete the WBI along with a few additional survey items between November of 2020 and 2021. Participation was voluntary and all responses were anonymous. Consent to participate was implied by completion of the survey. When this anonymous survey study was reviewed by the Mayo Clinic’s Institutional Review Board it was accordingly deemed not human subjects research, hence ethical approval was not required.

### Study measures

The conceptual model of how the clinical work system relates to burnout and professional well-being and its consequences, published in the National Academy of Medicine consensus study report, “Taking Action Against Clinician Burnout: A Systems Approach to Professional Well-being”, was used to inform the study measures [21]. The survey shown in the supplemental material (Table S1) asked participants their age, gender, years in current dental practice, primary practice setting, practice specialty, the 9-item WBI along with two additional items to assess burnout (from the Maslach Burnout Inventory, MBI), one item each to assess QOL and fatigue, and two items to assess adverse professional consequences (perceived major dental error and intent to leave their current job).

### Well-Being Index

The WBI is a 9-item screening instrument assessing burnout, stress, depression, mental and physical quality of life, fatigue, meaning in work, and work-life integration. The scoring algorithm for calculating WBI score is provided in Table S2. A higher WBI score indicates a greater level of distress in the participant. Multiple validation studies for the WBI have been conducted over the years among samples of over 29,300 healthcare professionals and general U.S. workers [32–38].

### Other measures

Two single items from the emotional exhaustion and depersonalization domains of the full MBI-HSS were used under license from Mind Garden, Inc. These two items have been shown to correlate well with the full emotional exhaustion and depersonalization domains of the MBI-HSS [39, 40]. Fatigue and overall QOL were measured using a single-item standardized linear analogue scale ranging from 0 to 10 respectively (0 = “As bad as it can be” and 10 = “As good as it can be”) [41–44]. A mean score was calculated among female and male responders. Participants who reported a symptom frequency of “weekly” or more often on either of the two domains were considered to have burnout. Suicidal ideation within the past 12 months was measured using an item from large US epidemiological studies [45, 46] and previous studies of physicians and nurses [16, 47]. Two additional items asked the dentists about their likelihood of leaving their current job in the next 24 months (response options: none, slight, definite, moderate, or likely) and concerns of having made a major dental error in the past three months using questions from previous studies of healthcare professionals [2, 10, 11].

### Relationship to other variables

Distress can manifest in many ways and there is no single definition for “severe distress”. Accordingly, we assessed the ability of WBI to:

1. Identify dentists with high overall QOL (well-being) as defined by a score of ½ SD above the gender matched mean for the group (a clinically meaningful effect size) [48].
2. Identify dentists with different manifestations of distress, including low overall QOL as defined by a score of ½ SD below the gender matched mean for the group (a clinically meaningful effect size); extreme fatigue as defined by having a fatigue score ½ SD worse than the gender matched mean for the group (a clinically meaningful effect size), and burnout.
3. Identify dentists who reported suicidal ideation in the past 12 months.
4. Stratify dentists’ likelihood of having made a major dental error in the past three months and reporting they intended to leave their current job within the next 24 months for reasons other than retirement.

### Statistical analysis

We utilized basic descriptive statistics and Fisher exact test or chi-square test to analyze the likelihood ratio (LRs), post-test probabilities, and univariate odds ratio associated with the WBI score for each of the outcomes, as appropriate. Wilcoxon, Kruskal-Wallis, or 2-sample *t* tests, were used to examine differences between groups. Sensitivity and specificity were calculated, and ROC curves were generated for the outcomes. We utilized SAS version 9 for all analyses.

## Results

The demographics and practice characteristics of the 597 participating dentists (estimated response rate 49.8%) are shown in Table 1. Almost all participants were general dentists (97.3%). Slightly over half of responders were male (53.9%). Nearly two-thirds of the participants were below the age of 45 years (64.6%). Most of the participants had been practicing dentistry for greater than five years (69.5%) and were associates in their respective practice groups (84.3%). The overall mean QOL score was 7.1 ± 1.6 (mean ± SD, range 0–10). Overall, 27.3% of dentists had low QOL and 46.1% had high QOL. A high level of fatigue was reported by 40.0% of dentists. Overall, 44.1% of dentist had burnout with 37.5% having high emotional exhaustion and 26.5% having high depersonalization. Among responding dentists, 6.2% reported suicidal ideation within the last year, 23.4% indicated a moderately or greater likelihood of leaving their current job for reasons other than retirement in the next 24 months, and 20.8% reported committing a major dental error within the last 3 months.

**Table 1:** Demographics of 597 Responders.

Variable	No. (%)
Gender	
Men	321 (53.9%)
Women	275 (46.1%)
Missing	1
Age	
<35	205 (34.3%)
35–44	181 (30.3%)
45–54	109 (18.3%)
55–64	70 (11.7%)
65+	32 (5.4%)
Years in current practice	
<5 Years	182 (30.5%)
5–14 Years	193 (32.3%)
15–24 Years	104 (17.4%)
25+ Years	118 (19.8%)
Current practice setting	
Associate (non-owner) - Dental Service Organization (DSO)	453 (75.9%)
Associate (non-owner) - Group private practice	50 (8.4%)
Partner - Group Private Practice	34 (5.7%)
Self-owned	58 (9.7%)
Other^a^	2 (0.3%)
Specialty	
General Dentist	578 (97.3%)
Other^b^	16 (2.7%)
Missing	3

^a^Other includes academic dentistry and public health dentistry.

^b^Other includes: Oral Implantologist (*n* = 3), Orofacial Pain (*n* = 1), Orthodontics and Dentofacial Orthopedics (*n* = 2), Pediatric Dentistry (*n* = 2), Periodontics (2), Prosthodontics (*n* = 4), and other (*n* = 2).

### WBI scores and ability to stratify QOL

**Fig. 1:**
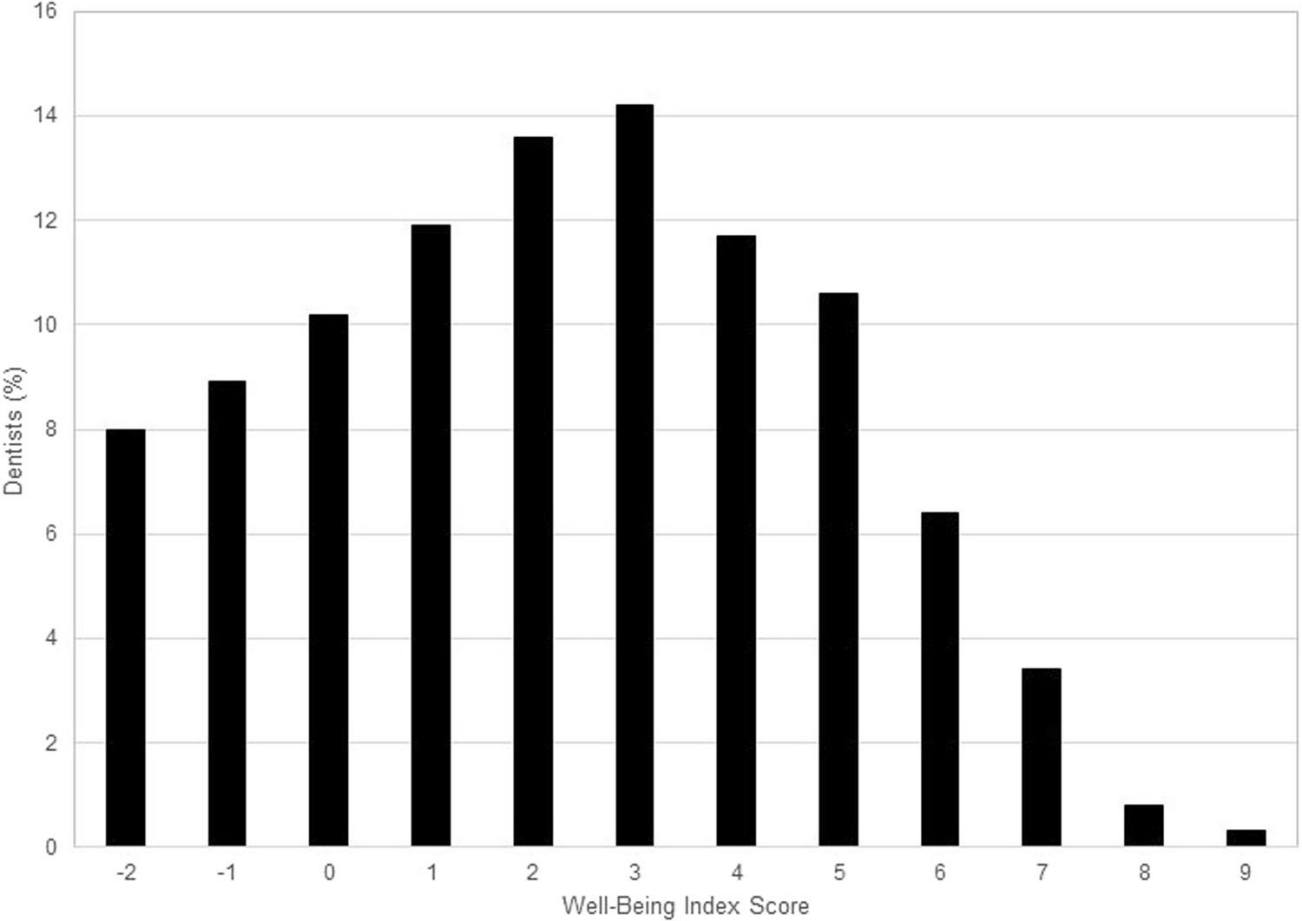
Well-Being Index scores among dentists. Bar graph representing the frequency of each WBI score in 597 U.S dentists. Lower scores are favourable while higher scores indicate greater levels of distress.

The overall mean WBI score was 2.3 ± 2.5 (mean ± SD, range −2 to 9). The frequency of each WBI score is shown in Fig. 1. The mean WBI score was statistically significantly greater in dentists with low QOL (4.1 ± 2.1) than among dentists without low QOL (1.6 ± 2.3, *P* < 0.001). The odds ratios of low versus high QOL ranged from 0.05 for those with a WBI score of −2 to 7.02 for those with a score of 7 or higher, indicating that the odds of a low QOL increased as the WBI score increased (Table 2). Based on an estimated pretest probability in the absence of a WBI score of 27.3% for low QOL, a WBI score of −2 lowered the posttest probability of low QOL to 2.1% and a WBI score of >7 raised it to 70.4% (Table 3). The area under the ROC curve of the WBI for low QOL was 0.79. The mean WBI score also proportionately related to high QOL. The odds of a high QOL increased with each decreasing WBI score (Table 3). The area under the ROC curve of the WBI for high QOL was 0.78.

**Table 2:** Well-Being Index Scores for Dentists with Low and High Overall QOL^a^.

WBI score	Low QOL				High QOL			
	Dentist with low overall QOL *N* = 163	Dentist wo. low overall QOL *N* = 434	OR (95% CI)	*p*-value	Dentist w. high overall QOL *N* = 275	Dentist wo. High overall QOL *N* = 322	OR (95% CI)	*p*-value
−2	1 (2.1%)	47 (97.9%)	0.05 (<0.01, 0.37)	0.003	39 (81.3%)	9 (18.8%)	5.75 (2.73, 12.10)	<0.001
−1	4 (7.5%)	49 (92.5%)	0.20 (0.07, 0.56)	0.002	43 (81.1%)	10 (18.9%)	5.78 (2.85, 11.75)	<0.001
0	4 (6.6%)	57 (93.4%)	0.17 (0.06, 0.47)	<0.001	45 (73.8%)	16 (26.2%)	3.74 (2.06, 6.79)	<0.001
1	10 (14.1%)	61 (85.9%)	0.40 (0.20, 0.80)	0.010	38 (53.5%)	33 (46.5%)	1.40 (0.85, 2.31)	0.18
2	14 (17.3%)	67 (82.7%)	0.51 (0.28, 0.94)	0.038	43 (53.1%)	38 (46.9%)	1.39 (0.87, 2.22)	0.17
3	27 (31.8%)	58 (68.2%)	1.29 (0.78, 2.12)	0.32	30 (35.3%)	55 (64.7%)	0.59 (0.37, 0.96)	0.032
4	25 (35.7%)	45 (64.3%)	1.57 (0.93, 2.65)	0.094	18 (25.7%)	52 (74.3%)	0.36 (0.21, 0.64)	<0.001
5	31 (49.2%)	32 (50.8%)	2.95 (1.73, 5.02)	<0.001	15 (23.8%)	48 (76.2%)	0.33 (0.18, 0.60)	<0.001
6	28 (73.7%)	10 (26.3%)	8.79 (4.16, 18.57)	<0.001	3 (7.9%)	35 (92.1%)	0.09 (0.03, 0.30)	<0.001
≥7	19 (70.4%)	8 (29.6%)	7.02 (3.01, 16.39)	<0.001	1 (3.7%)	26 (96.3%)	0.04 (<0.01, 0.31)	0.002

^a^Low overall QOL is defined by a score 1/2 standard deviation (SD) below the mean for the overall population. High overall QOL is defined by a score 1/2 standard deviation (SD) above the mean for the overall population.

**Table 3:** Ability of the Well-Being Index to Identify Quality of Life and Distress among Dentists.

WBI Score	Low overall QOL		High overall QOL		High Fatigue		Burnout		Suicidal Ideation^a^	
	LR^b^	Post test prob^c^	LR	Post test prob	LR	Post test prob	LR	Post test prob	LR	Post test prob
−2	0.06 (0, 0.42)	2.1%	5.07 (1.96, 14.68)	80.6%	0.23 (0.06, 0.73)	10.4%	0.03 (0, 0.20)	2.1%	0.13 (0.31, 0.49)	0.9%
−1	0.22 (0.05, 0.73)	7.5%	5.03 (2.05, 13.66)	80.5%	0.16 (0.03, 0.55)	7.5%	0.10 (0.02, 0.35)	7.5%		
0	0.19 (0.04, 0.61)	6.6%	3.29 (1.53, 7.42)	73.0%	0.35 (0.13, 0.85)	14.8%	0.14 (0.04, 0.39)	9.8%		
1	0.44 (0.17, 1.01)	14.1%	1.35 (0.71, 2.58)	52.5%	0.59 (0.27, 1.21)	22.5%	0.43 (0.20, 0.87)	25.3%		
2	0.56 (0.25, 1.15)	17.3%	1.32 (0.73, 2.41)	52.1%	1.12 (0.60, 2.06)	35.8%	0.83 (0.45, 1.51)	39.5%	1.00 (0.27, 2.65)	6.2%
3	1.24 (0.66, 2.25)	31.8%	0.64 (0.35, 1.16)	34.4%	1.22 (0.67, 2.18)	37.7%	1.36 (0.76, 2.43)	51.8%	1.15 (0.36, 2.83)	7.1%
4	1.48 (0.75, 2.84)	35.7%	0.41 (0.19, 0.82)	24.9%	1.19 (0.60, 2.29)	37.1%	3.17 (1.59, 6.57)	71.4%	1.95 (0.70, 4.45)	11.4%
5	2.58 (1.30, 5.08)	49.2%	0.37 (0.16, 0.79)	23.1%	2.22 (1.11, 4.41)	52.4%	5.40 (2.39, 13.21)	81.0%	1.89 (0.62, 4.60)	11.1%
6	7.46 (2.79, 21.49)	73.7%	0.10 (0.02, 0.41)	7.6%	2.77 (1.10, 7.08)	57.9%	6.77 (2.19, 25.29)	84.2%	0.84 (0.08, 4.01)	5.3%
>7	6.32 (1.99, 22.03)	70.4%	0.05 (0, 0.38)	3.6%	8.87 (2.45, 40.03)	81.5%	15.87 (2.91, 188.87)	92.6%	5.30 (1.46, 16.02)	25.9%

We defined (1) high or low overall QOL as having a standardized linear analog QOL score of more than 0.5 SD above, or 0.5 SD or less below, that of the gender matched mean for the groups, respectively; (2) extreme fatigue as having a standardized linear analog score of 0.5 SD or more below that of the gender matched mean for the group (high score is favorable); and (3) burnout as having high emotional exhaustion or high depersonalization on the Maslach Burnout Inventory items.

^a^For suicidal ideation, scores of −2 to 1 were combined due to small number of events that occurred among responders with these scores. The LR and posttest probabilities reported are for the group of responders with a WBI score between −2 and 1.

^b^LR indicates the likelihood ratio associated with the WBI exact score.

^c^Posttest probability was calculated using an estimated prevalence of 27.3% for low overall QOL, 45.1% for high overall QOL, 33.2% for extreme fatigue, 44.1% for burnout, and 6.2% for suicidal ideation as the pretest probability.

### Ability of WBI detect extreme fatigue, burnout, and suicidal ideation

Dentists with extreme fatigue, burnout, and suicidal ideation had statistically significantly higher mean WBI scores than those without extreme fatigue (3.6 ± 2.3 vs 1.6 ± 2.4; *P* < 0.001), burnout (4.0 ± 2.0 vs 1.0 ± 2.1; *P* < 0.001) or suicidal ideation (4.2 ± 2.2 vs 2.1 ± 2.5; *P* < 0.001). As the WBI score increased so did the odds of extreme fatigue (score of 0, OR 0.21; score of ≥7, OR 8.20), burnout (score of 0, OR 0.02; score of ≥7, OR 12.20) and suicidal ideation (score of 0, OR 0.30; score of ≥7, OR 15.53). Assuming a prevalence of 33.2% as the pretest probability for extreme fatigue, the WBI lowered the posttest probability to 10.4% or raised it to 81.5% across its range (Table 3). Assuming a prevalence of 44.1% as pretest probability for burnout, the WBI lowered the posttest probability to 2.1% or raised it to 92.6% across its range. Similarly, assuming a prevalence of 6.2% as the pretest probability for suicidal ideation, the WBI lowered the posttest probability to 0.9% or raised it to 25.9% across its range. The area under the ROC curve of the WBI for extreme fatigue, high burnout, and suicidal ideation was 0.72, 0.84, and 0.73, respectively.

### Association between WBI score and perception of recent major dental error and intent to leave the current job

Dentists who reported a major dental error in the past three months reported a statistically significantly higher mean WBI score than those who reported no such concerns (3.4 ± 2.3 vs 2.0 ± 2.5; *P* < 0.001). Using a prevalence of 20.1% as the pretest probability for concerns regarding major dental errors, the WBI lowered the posttest probability to 6.0% or raised it to 32.4% across range. Dentists who reported an intent to leave their current job within 24 months had a statistically significantly higher mean WBI score than those who reported no such intent (3.9 ± 2.2 vs 1.8 ± 2.4; *P* < 0.001). Using a prevalence of 23.5% as the pretest probability for moderate or higher intent to leave current job, the WBI lowered the posttest probability to 4.2% or raised it to 55.6% across its range (Table 4). The area under the ROC curve of the WBI for concerns regarding a major dental error and for intent to leave current job and was 0.66 and 0.73, respectively.

**Table 4:** Ability of the Well-Being Index to Identify a Recent Major Dental Error and Moderate or Higher Intent to Leave the Current Job.

WBI Score	Dental Error		Moderate or Higher Intent to Leave	
	LR^a^	Post test prob^b^	LR	Post test prob
−2	0.25 (0.04, 0.98)	6.0%	0.14 (0.01, 0.68)	4.2%
−1	0.40 (0.10, 1.21)	9.1%	0.13 (0.01, 0.60)	3.8%
0	0.27 (0.06, 0.87)	6.3%	0.29 (0.07, 0.87)	8.2%
1	0.94 (0.41, 1.97)	19.0%	0.73 (0.31, 1.57)	18.3%
2	0.94 (0.44, 1.87)	19.1%	0.93 (0.45, 1.82)	22.2%
3	1.33 (0.69, 2.47)	25.1%	0.76 (0.36, 1.50)	18.8%
4	1.22 (0.57, 2.47)	23.5%	1.70 (0.86, 3.27)	34.3%
5	1.53 (0.71, 3.13)	27.7%	2.29 (1.14, 4.51)	41.3%
6	2.77 (1.09, 6.84)	41.1%	3.26 (1.30, 8.08)	50.0%
≥7	1.91 (0.57, 5.87)	32.4%	4.08 (1.35, 12.50)	55.6%

^a^LR indicates the likelihood ratio associated with the WBI exact score.

^b^Posttest probability was calculated using an estimated prevalence of 20.1% for dental error, 23.5% for moderate or higher intent to leave.

### Threshold score

A WBI score of 3 or more was found to be a meaningful threshold to identify dentists at increased risk (LR > 1) for several adverse outcomes. Overall, 47.4% of the responding dentists had a score of ≥3. Dentists with scores of ≥3 had higher likelihoods of low QOL LR 2.26 [95% CI, 1.82–2.78], extreme fatigue (LR 1.84 [95% CI, 1.46–2.31]), burnout (LR 3.17 [95% CI, 2.44–4.14]) and suicidal ideation (LR 1.79 [95% CI, 1.31–2.24]) than dentists with scores 2 and lower. A threshold score of 3 or more was also associated with increased likelihood of reporting a recent major dental error (LR 1.56 [95% CI, 0.69–2.47]) and intent to leave current job (LR 1.78 [95% CI, 1.41–2.22]).

## Discussion

To our knowledge, this is the first study that evaluates the validity evidence for a composite measure of well-being among US dentists. Among the sample of almost 600 dentists, the WBI identified distress across a spectrum of domains (low QOL, extreme fatigue, burnout, and suicidal ideation) as well as well-being (high QOL). Furthermore, the WBI score stratified dentists’ likelihood of adverse professional consequences as indicated by their intent to leave their current job and by their concern for having committed a major dental error. These findings suggest that the WBI can be an effective tool in identifying various manifestations of distress and well-being among dentists and predicting relevant outcomes.

In this cohort of dentists, the mean WBI score and score distribution was similar to a 2019 cohort of pharmacists and less favorable than WBI scores reported in a 2012 cohort of physicians, 2016 cohort of advanced practice professionals (e.g., physician assistants, nurse practitioners) and 2012 cohort of other US workers. Given the timespan across these studies and the COVID-19 pandemic, the significance of the differences in WBI score across health worker populations is unknown and merits further investigation [33, 34, 38]. We did find, however, that WBI stratified risk comparable with previous studies in other healthcare workers [32–34]. A substantial proportion of dentists in this cohort had high levels of fatigue, placing them at risk for potentially serious health consequences [49, 50]. More than 1 in 17 participants reported suicidal ideation in the past year. This is similar to previous findings among other healthcare workers in United States [16, 47]. Since as early as the 1960s, a higher rate of suicide has been speculated among dentists relative to other professions. However, systematic evaluation of suicidal ideation or suicidal rates among dentists is lacking, and the available limited data is inconsistent and outdated [28, 51, 52]. Suicidal ideation is a well-established precursor to suicidal attempts and death by suicide. The high prevalence of recent suicidal ideation in this cohort of dentists is concerning and would benefit from additional research.

Among the dentists in this cohort, 20.8% reported having made a recent major dental error within the last 3 months. Dentists with a WBI score of 3 or higher had 56% higher likelihood of reporting they had recently made such an error. The WBI stratified dentists’ likelihood of reporting they had made a recent major dental error. This suggests a relationship between severity of distress among dentists, as detected by the WBI, and negative patient outcomes. This finding is consistent with previously correlated data between burnout and concerns of major dental error reported by dentists [53]. Additionally, nearly a quarter of the dentists in this study were at least moderately likely to leave their current job for reasons other than retirement in the next 24 months. The higher the WBI score, the higher the odds of a dentist considering leaving their current practice. Despite the high prevalence of distress, nearly half of participating dentists had high QOL and the WBI was able to stratify individuals across the positive well-being spectrum.

Given the ability of the WBI to identify various manifestations of distress and well-being and stratify risk of relevant outcomes, the WBI may be a useful tool to improve dentists’ well-being both at an individual as well as at the organizational level. Previous research suggests that providing healthcare professionals objective, individualized feedback on their results immediately after completing the WBI can help foster behavioral change to promote well-being [29]. The WBI may be valuable to dentists who are doing well, as a way of validating their perceptions and encouraging them to continue engaging with well-being strategies and remain in work environments that are serving them well. On the other end of the spectrum, dentists whose WBI score indicates low well-being may be prompted to act, including help-seeking and accessing resources such as the 2021 ADA dentist well-being resources [26]. The WBI may also be useful at an organizational level where continued assessment of well-being among dentists can provide crucial insights into the impact of current workload and workflows on dentists’ well-being. Given the relationship between WBI scores and dentists’ well-being as well as their likelihood of reporting they had made a recent major dental error and intending to leave their current practice, periodic assessment of well-being among dentists, followed by timely interventions, may be a crucial step in improving patient safety, healthcare outcomes, and retention.

There are several limitations to the present study. Owing to the varied channels of survey promotion, survey response rate could only be estimated, and response bias may impact the results. The survey was predominantly completed by non-owner general dentists affiliated with a Dental Service Organization or a group practice, so dentists who are practice owners or those in specialty practices are not adequately represented in this study, potentially limiting the generalizability of the findings to those populations. While the gender distribution among the survey participants was comparable to U.S dentists nationally, participants were generally younger [26]. It is unknown whether dentists experiencing distress would be more likely to participate due to interest in the topic or less likely to participate because they are disengaged or overwhelmed. Previous studies of non-responders in surveys evaluating healthcare professional well-being have found similar rates of burnout in those who were initially non-responders and suggest participants are generally representative of the overall sample [54–56]. Although the WBI screens for multiple dimensions of distress, the WBI does not evaluate all manifestations of distress. Additionally, the WBI is not designed to evaluate or diagnose any mental health condition or specifically screen for suicidal ideation. Thus, individuals who score high benefit from professional evaluation. Finally, this study is cross-sectional and no causality or directionality of effects can be deduced.

## Conclusion

In conclusion, the WBI identified distress across multiple dimensions including low QOL, extreme fatigue, burnout, and suicidal ideation among dentists, as well as well-being. Moreover, an elevated WBI score correlated with increased risk for adverse professional consequences. Therefore, the WBI can be an effective screening tool to identify distress and evaluate well-being in dentists. Additional research dedicated toward further understanding the various contributing factors influencing dentists’ well-being and defining organizational strategies to address dentists’ well-being is warranted.

### Supplementary information

Supplementary Information

## Data Availability

The participants of this study did not give written consent for their data to be shared publicly, so due to the sensitive nature of the research supporting data is not available.
