# Supplementary material for: Distress and well-being in dentists: performance of a screening tool for assessment
Source: BDJ Open. 2024 Jan 16;10:3. doi: 10.1038/s41405-024-00185-9 (PMC10791682; doi:10.1038/s41405-024-00185-9)
Supplement: Supplementary file 1 — Supplementary Information [file 41405_2024_185_MOESM1_ESM.pdf]

Table S1:

Survey Items

1. What is your gender
    a. Male
    b. Female
    c. No answer
    d. Gender diverse
2. What is your age
    a. Less than 35
    b. 35-44
    c. 45-54
    d. 55-64
    e. 65 or older
3. What is your practitioner type?
    a. Dental hygienist
    b. Dentist
4. (3a) Please indicate your highest level of training related to being a dental hygienist
    a. Associate
    b. Bachelor of Science in Dental Hygiene
    c. Dual degree B.S/M.S
    d. Master of Science Dental Hygiene.
5. (3b) What type of dentist are you?
    a. General dentist
    b. Endodontics
    c. Oral and Maxillofacial Pathology
    d. Oral and Maxillofacial Radiology
    e. Oral and Maxillofacial Surgery
    f. Oral Implantologist
    g. Oral Medicine
    h. Orofacial Pain
    i. Orthodontics and Dentofacial Orthopedics
    j. Pediatric Dentistry
    k. Periodontics
    l. Prosthodontics

    m. Other
6. How many years have you been a practicing dentist or dental hygienist?
    a. Less than 5 years
    b. 5-14 years
    c. 15-24 years
    d. 25 years or longer
7. What is your primary practice setting?
    a. Self-owned solo private practice
    b. Associate (non-owner) in group private practice (single location dental practices with multiple dentists practicing in that one location)
    c. Associate (non-owner) in a group dental support/service organization (DSO, support for non-clinical functions are centralized in one location for several offices)
    d. Partner in a group private practice
    e. Academic dentistry
    f. Public health dentistry
    g. Hospital dentistry
    h. Military/veterans administration hospital, clinic, etc.
8. How would you describe your overall quality of life?
    a. Response option 0 to 10
9. How would you describe your level of fatigue on average
    a. Response option 0 to 10
10. During the past 12 months have you had thoughts of taking your own life?
    a. Yes
    b. No
11. Are you concerned you have made a major dental error in the last 3 months?
    a. Yes
    b. No
12. What is the likelihood that you will leave your current job situation within 2 years for reasons other than retirement?
    a. None
    b. Slight
    c. Definite
    d. Moderate
    e. Likely
13. About how many hours altogether did you work in the past 7 days?
14. & 15. Two items from Maslach Burnout Inventory used under license.

Table S2

WBI Scoring

To score the 9-item WBI, one point is assigned for each “yes” response to the original 7-item WBI. For the meaning in work item (“The work I do is meaningful to me”), one point is added for unfavorable responses indicating less meaning in work (one or two on the seven-item scale), one point is subtracted for favorable responses (six or seven on the seven-item scale), and no points are assigned for other responses (three, four, or five on the seven-item scale). For the work-life integration item (“My work schedule leaves enough time for my personal/family life”), one point is added for unfavorable responses indicating lower satisfaction (disagree or strongly disagree), whereas one point is subtracted for favorable responses (agree or strongly agree). The total WBI score therefore yields a 12 point range from -2 to 9, with higher scores indicating a greater extent of distress.

Scoring of WBI

	**Points assigned**
1. have you felt burned out from your work?	1
2. have you worried that your work is hardening you emotionally?	1
3. have you often been bothered by feeling down, depressed, or hopeless?	1
4. have you fallen asleep while sitting inactive in a public place?	1
5. have you felt that all things you had to do were piling up so high that you could not overcome them?	1
6. have you been bothered by emotional problems (such as feeling anxious, depressed, or irritable)?	1
7. has your physical health interfered with your ability to do your daily work at home and/or away from home?	1
8. The work I do is meaningful to me	
response option of a 1 or 2	1
response option of 3 to 5	0
response option of 6 or 7	-1
9. My work schedule leaves me enough time for my personal/family life	
disagree; strongly disagree	1
neutral	0
agree, strongly agree	-1

The WBI instrument is copyrighted, and permission for use can be obtained by contacting the team at www.MyWellBeingIndex.org
